# Potential options for managing LOX+ ER− breast cancer patients

**DOI:** 10.18632/oncotarget.9073

**Published:** 2016-04-28

**Authors:** Yong Han, Shenyi Lian, Xingran Cui, Kexin Meng, Balázs Győrffy, Tao Jin, Dongsheng Huang

**Affiliations:** ^1^ Clinical Research Institute, Zhejiang Provincial People's Hospital, Hangzhou, China; ^2^ Key Laboratory of Carcinogenesis and Translational Research (Ministry of Education), Department of Biochemistry and Molecular Biology, Peking University Cancer Hospital and Institute, Beijing, China; ^3^ Division of Interdisciplinary Medicine and Biotechnology, Department of Medicine, Beth Israel Deaconess Medical Center/Harvard Medical School, Boston, Massachusetts, USA; ^4^ Department of Thyroid Breast Surgery, Zhejiang Provincial People's Hospital, Hangzhou, China; ^5^ Momentum Cancer Biomarker Research Group, Research Centre for Natural Sciences, Hungarian Academy of Sciences, Budapest, Hungary; ^6^ Second Department of Pediatrics, Semmelweis University, Budapest, Hungary; ^7^ Department of Cardiothoracic Surgery, Zhejiang Provincial People's Hospital, Hangzhou, China

**Keywords:** LOX, estrogen recepter, EMT, chemoresistance, bisphosphonates

## Abstract

Overexpression of lysyl oxidase (LOX) is often observed in estrogen receptor negative (ER–) breast cancer patients with bone metastasis. In the present bioinformatics study, we observed that LOX is a prognostic factor for poor progression free survival in patients with ER– breast cancer. LOX overexpression was positively correlated with resistance to radiation, doxorubin and mitoxantrone, but negatively correlated with resistance to bisphosphonate, PARP1 inhibitors, cisplatin, trabectedin and gemcitabine. LOX overexpression was also associated with EMT and stemness of cancer cells, which leads to chemotherapeutic resistance and poor outcome in ER– patients. Although we suggest several therapeutic interventions that may help in the management of LOX+ ER– breast cancer patients, experiments to validate the function of LOX in ER– breast cancer are still needed.

## INTRODUCTION

Breast cancers in which the tumor is estrogen receptor (ER)-negative (ER–) account for approximately 30% of white patients [1] and about 40%–50% of Chinese patients [2]. These patients tend to have a poorer prognosis with a higher risk of disease recurrence and metastasis than patients with ER+ tumors. This is in part because there are fewer effective methods for preventing and treating ER– breast cancers. In addition, the molecular subtypes of ER– tumors are not well defined due to their biological heterogeneity. In recent years, however, a number of potential signaling pathways driving ER– breast cancer have been identified [3, 4]. This makes targeted therapy focusing on a specific molecular subtype a potentially effective strategy for managing ER– breast cancer patients. That said, the clinical application of such new approaches remains a prospect for the future.

Lysyl oxidase (LOX) is an enzyme involved in regulating extracellular matrix (ECM) and connective tissue homeostasis [5]. Moreover, previous studies have shown that LOX plays a crucial role in mediating proliferation, migration, and invasion by endometrial and endometriotic cells. Specifically, LOX influences the expression of genes related to fibrosis and ECM remodeling, including E-cadherin [6], which is indicative of its pro-metastatic potential. A recent study by Thomas et al. [7] showed that LOX induces pre-metastatic bone lesions in breast cancer patients (especially ER– subgroup) by disrupting normal bone homeostasis. These lesions subsequently facilitate colonization by circulating tumor cells (CTCs), leading to bone metastasis. The authors of that report suggest administration of bisphosphonate to breast cancer patients with LOX overexpression may prevent establishment and proliferation of CTCs within bone. Nevertheless, the molecular mechanisms underlying LOX-promoted ER– breast cancer metastasis and the best approach to treating LOX+ ER– breast cancer patients remains unknown.

To promote translational research on LOX towards greater clinical significance, in this study, we address the following questions: how is LOX associated with metastasis and estrogen receptor 1 (ESR1) expression; why does bisphosphonate inhibit metastasis in patients overexpressing LOX; and are there any promising therapeutic options for managing LOX+ ER– breast cancer patients.

## RESULTS

### LOX overexpression is associated with poor PFS and metastasis

**Figure 1:**
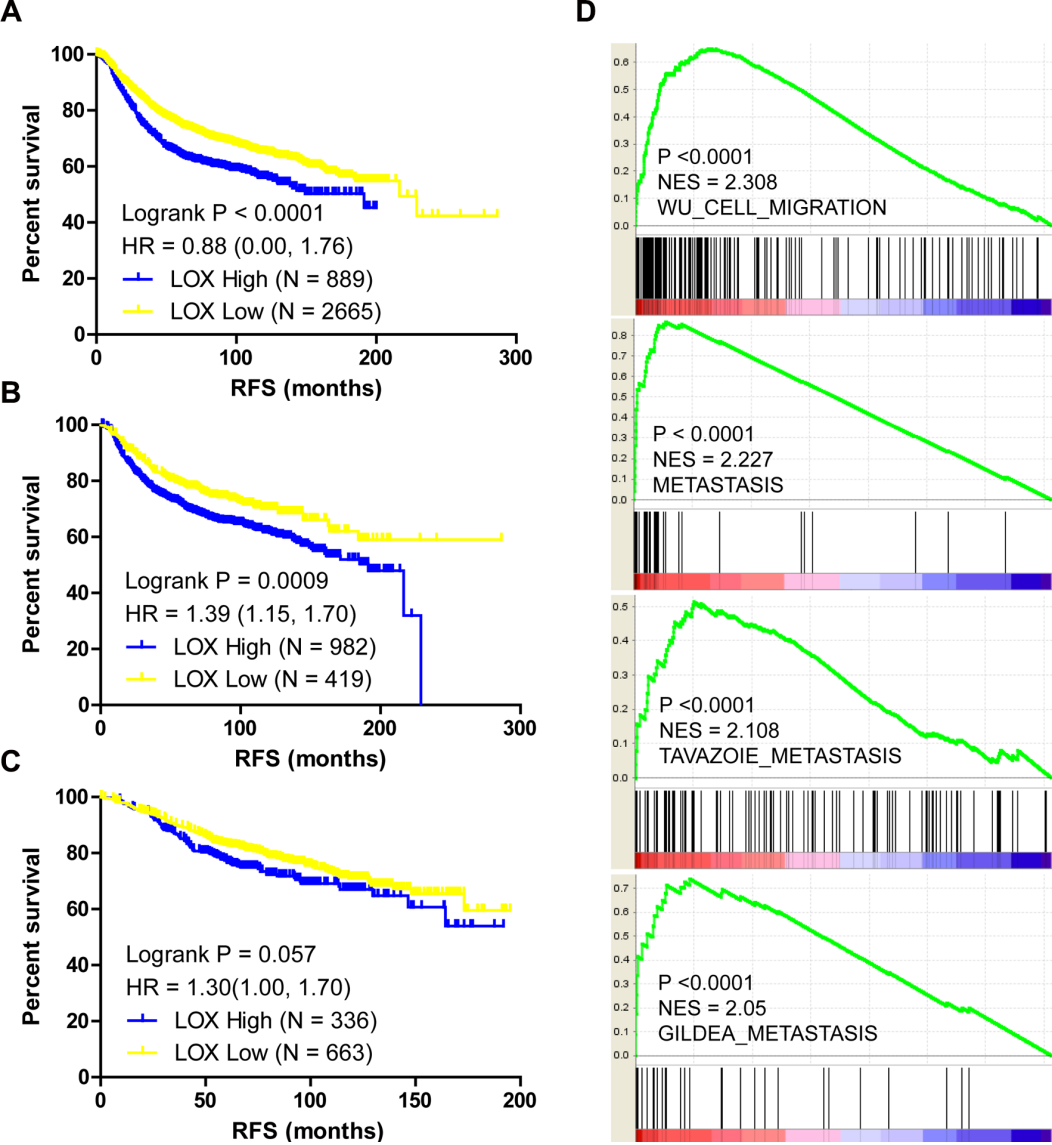
LOX expression correlates with PFS and metastasis in breast cancer patients LOX expression correlates negatively with the PFS among all breast cancer patients (**A**) and among ER– breast cancer patients (**B**). (**C**) There is no significant correlation between LOX expression and PFS among ER+ breast cancer patients. (**D**) GSEA analysis indicating that LOX expression correlates positively with migration and metastasis.

**Figure 2:**
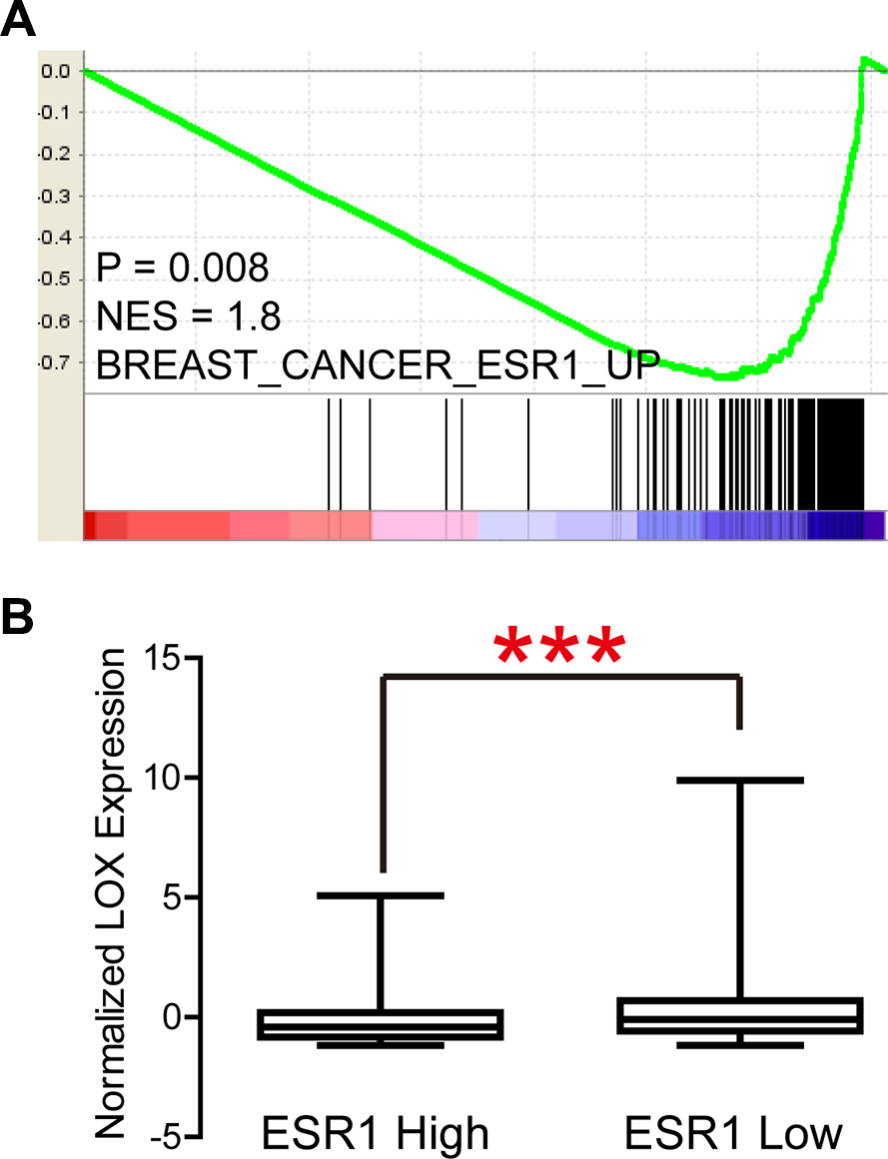
LOX expression correlates negatively with ESR1 signatures (**A**) and ESR1 expression (**B**).

LOX overexpression is reported to be specifically associated with bone relapse in ER– breast cancer patients [7]. In the present study, we analyzed the Gyorffy dataset to explore the relation between LOX and progression free survival (PFS) in breast cancer patients. We found that LOX is associated with PFS among all patients (Figure 1A, *P* < 0.0001) and ER– patients (Figure 1B, *P* = 0.0009), but not ER+ patients (Figure 1C, *P* > 0.05). Furthermore, the results of gene set enrichment analysis (GSEA) indicate that LOX expression correlates positively with gene sets that represent cancer cell migration and metastasis in breast cancer (Figure 1D, *P* < 0.0001). GSEA also revealed that LOX expression correlates negatively with the ESR1 gene signature (Figure 2A, *P* = 0.008) and that LOX expression is significantly higher in ESR1-low than ESR1-high patients (Figure 2B). These results indicate that LOX is a strong predictor of poor PFS in ER– breast cancer patients and is closely associated with metastasis.

### Bisphosphonate is a therapeutic option for LOX+ breast cancer patients

**Figure 3:**
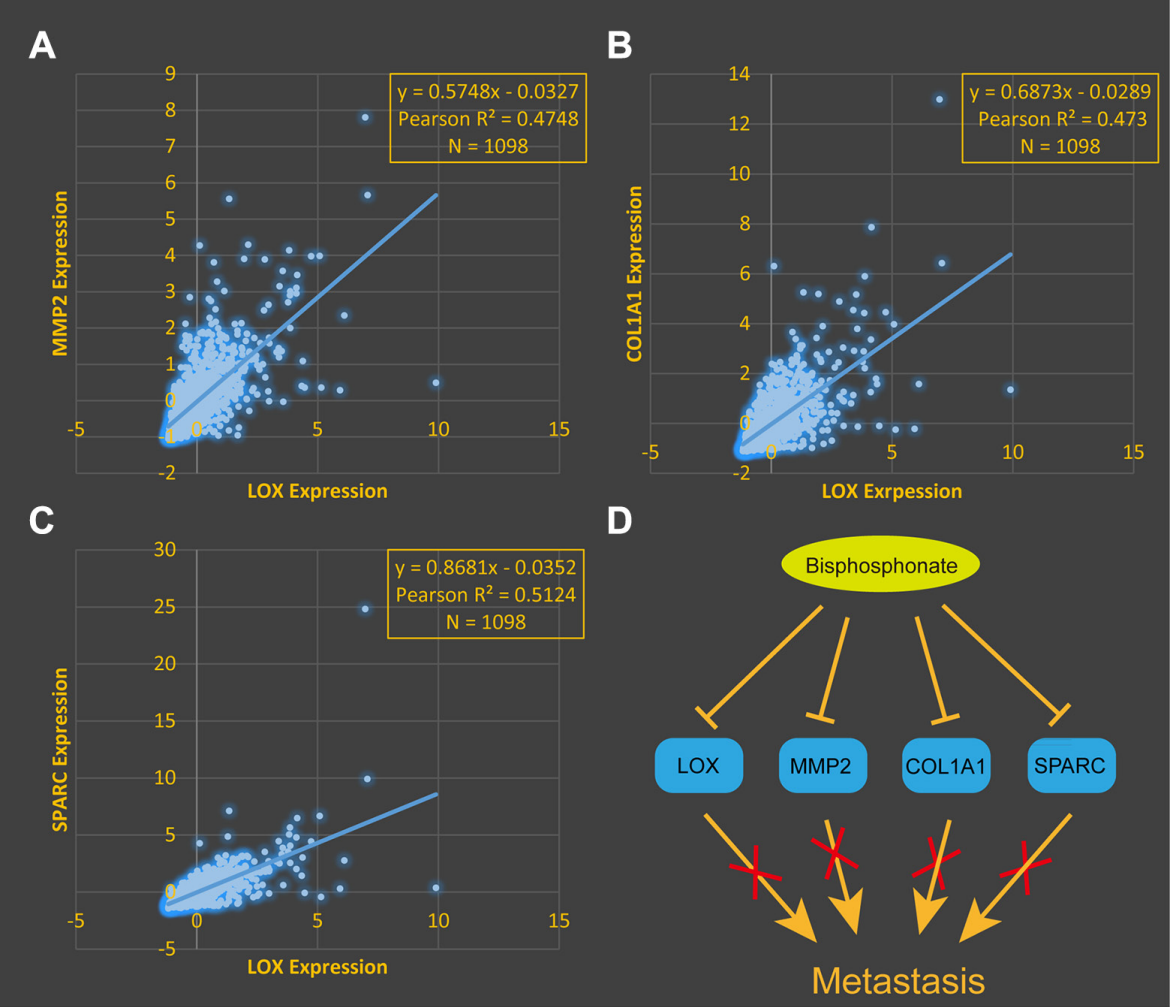
LOX expression correlates positively expression of MMP2 (**A**), COL1A1 (**B**) and SPARC (**C**). (**D**) Diagram summarizing results indicating bisphosphonate suppresses metastasis by inhibiting LOX, MMP2, COL1A1 and SPARC expression.

Bisphosphonate treatment may suppress bone metastasis in ER– breast cancer patients overexpressing LOX [7]. To explore the underlying mechanism, we reanalyzed breast cancer data from The Cancer Genome Atlas, which revealed that LOX expression correlates with expression of matrix metallopeptidase 2 (MMP2) (Figure 3A, R^2^ = 0.47), collagen type I alpha1 (COL1A1) (Figure 3B, R^2^ = 0.47), and secreted protein acidic and rich in cysteine (SPARC) (Figure 3C, R^2^ = 0.51). Given that MMP2, COL1A1 and SPARC are all pro-metastatic genes [8–10], we suggest these genes play crucial roles in LOX+ breast cancer metastasis. In addition, data mining results from The Comparative Toxicogenomics Database indicates that bisphosphonate down-regulates expression of LOX, MMP2, COL1A1 and SPARC, which means bisphosphonate may suppress cancer metastasis by targeting these four genes.

### Unfavorable characteristics correlated with LOX overexpression

**Figure 4:**
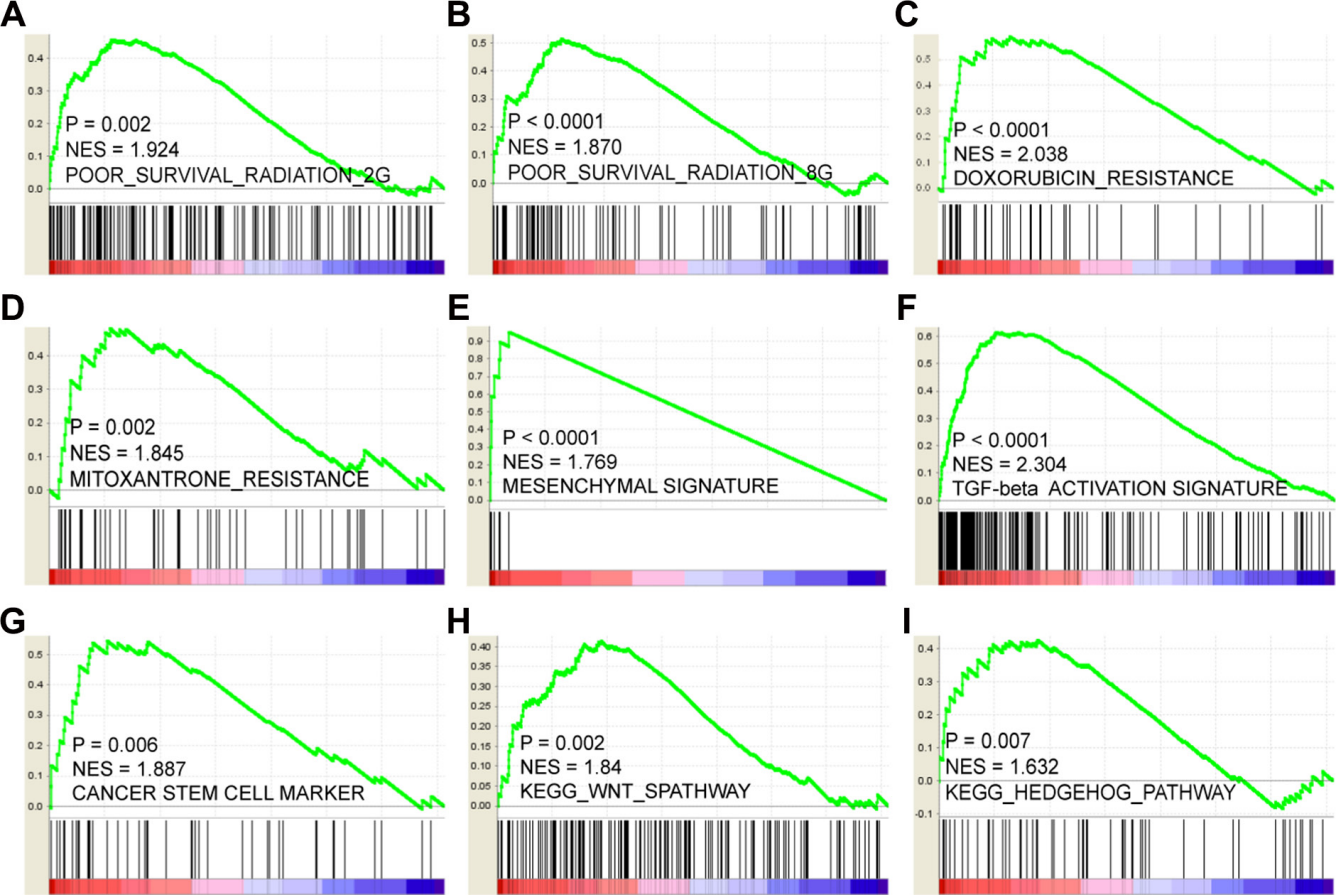
LOX correlates positively with resistance to radiation therapy (**A** and **B**), doxorubicin (**C**) and mitoxantrone (**D**). LOX expression also correlates with mesenchymal gene expression signature (**E**) and TGF-β pathway activation (**F**). High levels of LOX expression correlates with overexpression of cancer stem cell markers (**G**) and activation of stem cell pathways such as the WNT (**H**) and HEDGEHOG (**I**) signaling pathways.

We next sought to explore the mechanisms and characteristics underlying the LOX-associated poor PFS. GSEA results showed that LOX expression positively correlates with gene signatures that represent poor outcome after radiation therapy. This finding indicates that LOX+ breast cancer patients are resistant to radiation therapy (Figure 4A and 4B). Similarly, it may not be a good choice for LOX+ patients to choose doxorubicin (Figure 4C) or mitoxantrone (Figure 4D) for chemotherapy. Other analyses indicated that LOX overexpression correlates with epithelial-mesenchymal transition (EMT) (Figure 4E and 4F) and activation of cancer stem cell pathways, such as the WNT and HEDGEHOG pathways (Figure 4G–4I). Thus, resistance to radiation and certain drugs, EMT transition, and harboring cancer stem cell like characteristics may contribute to the LOX-related poor prognosis.

### Favorable characteristics correlated with LOX overexpression

**Figure 5:**
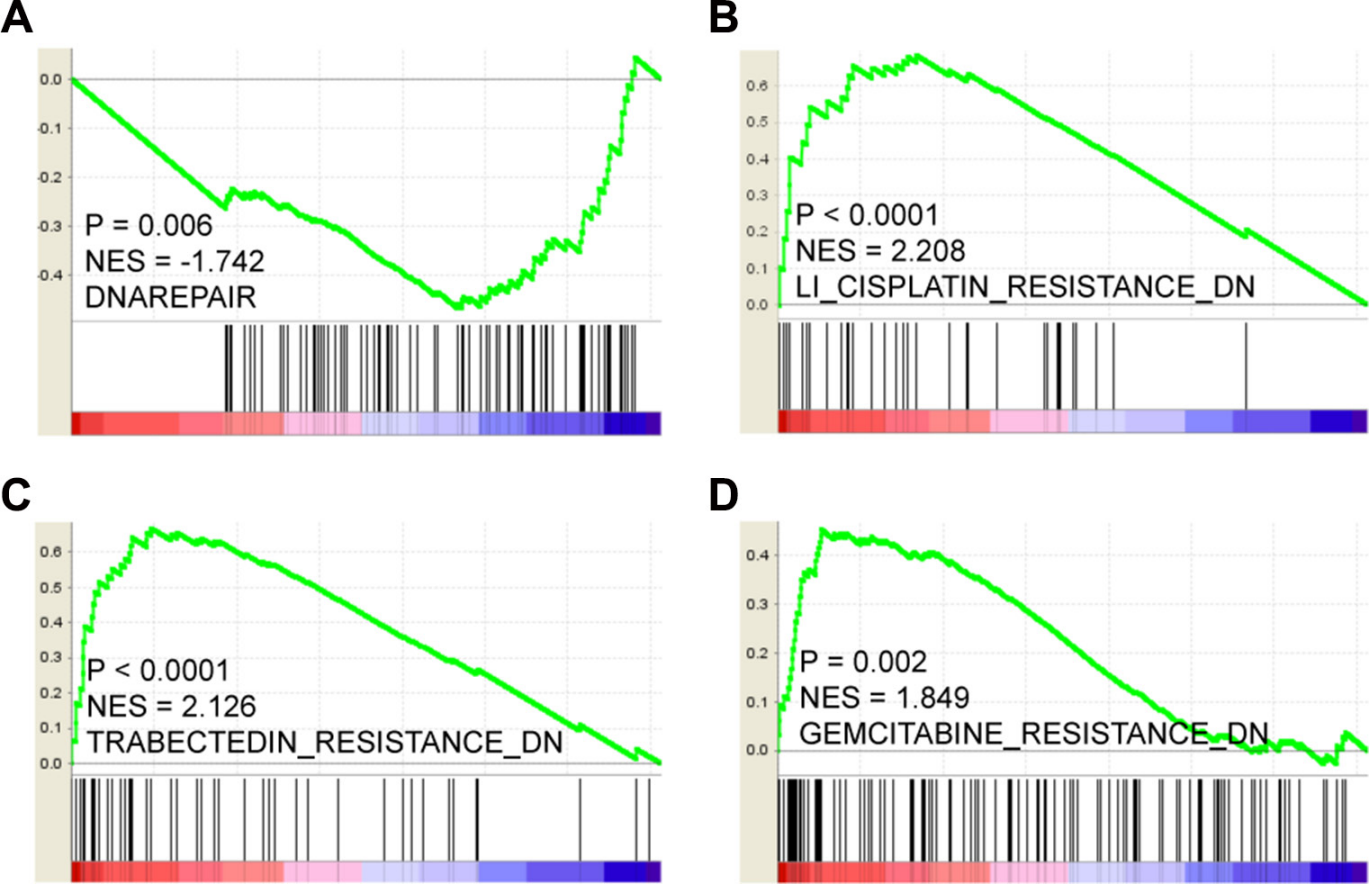
(**A**) LOX expression correlates negatively with DNA repair gene expression and with down-regulation of genes involved in cisplatin (**B**), trabectedin (**C**) and gemcitabine (**D**) resistance processes.

**Figure 6:**
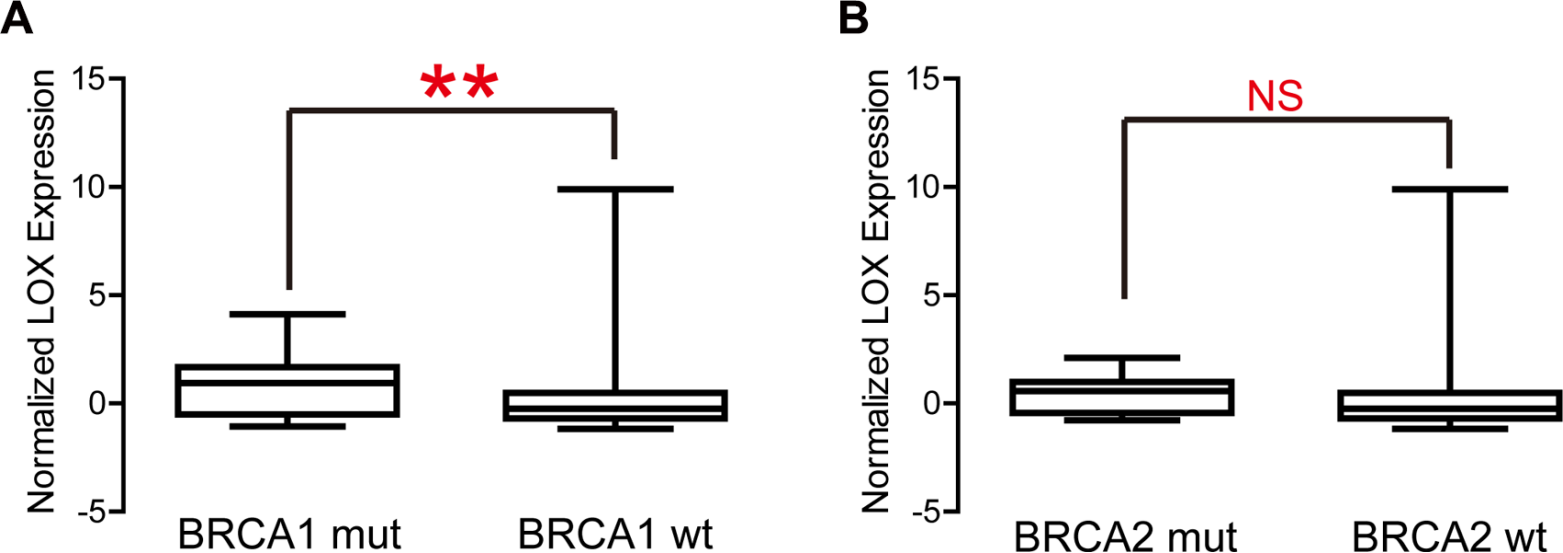
LOX expression is significantly higher in BRCA1 mutation carriers than the wild type group (**A**), whereas there is no difference between BRCA2 mutation carriers and the BRCA2 wild type group.

Overexpression of LOX does present certain advantages. For instance, LOX expression correlates negatively with expression of genes associated with DNA repair (Figure 5A, *P* = 0.006), but correlates positively with genes down-regulated in samples resistant to cisplatin, trabectedin and gemcitabine (Figure 5B–5D, *P* < 0.0001, *P* < 0.0001, *P* = 0.002, respectively). This indicates that cytotoxic drugs such as cisplatin and gemcitabine will likely achieve a better clinical response in patients overexpressing LOX. In addition, LOX expression is significantly higher among carriers of BRCA1 mutations than among those without BRCA1 mutation (Figure 6A), though LOX expression does not differ between BRCA2 mutation and wild type carriers (Figure 6B). Since poly (ADP-ribose) polymerase (PARP) inhibitors are effective for treatment of breast cancer patients with BRCA1 mutation [11], this class of drugs may also be effective for managing breast cancer patients who overexpress LOX and carry BRCA1 mutation.

### Appropriate therapeutic options for LOX+ ER– breast cancer patients

**Figure 7:**
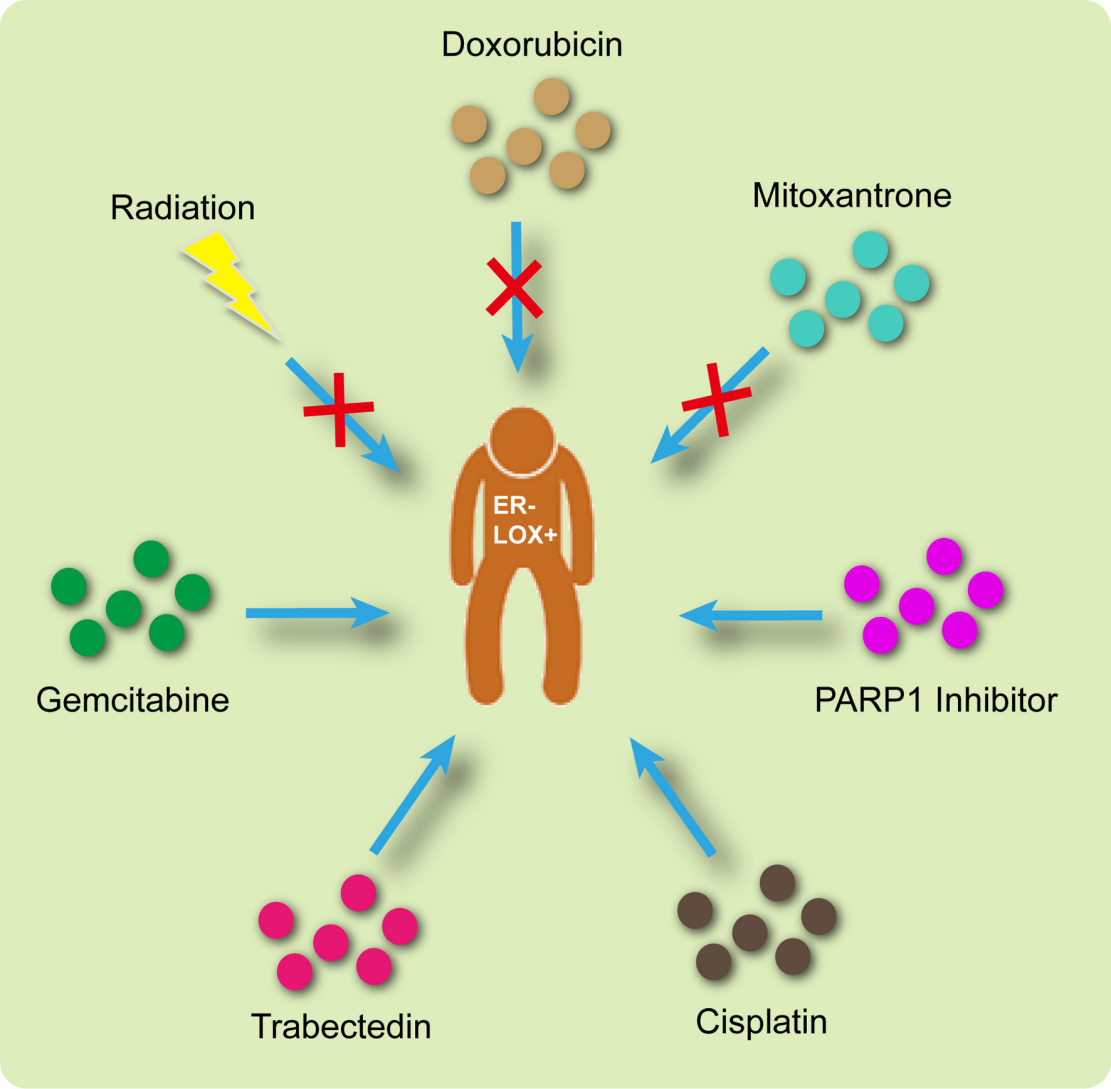
Appropriate therapeutic interventions for ER– LOX+ breast cancer patients

Based on the analysis summarized above, the appropriate treatment options are illustrated in Figure 7. Briefly, radiation, doxorubicin and mitoxantrone would be ineffective in LOX+ ER– patients. On the other hand, a PARP1 inhibitor, cisplatin, trabectedin and gemcitabine may produce promising results.

## DISCUSSION

The ER– breast cancer subtype is associated with a significantly higher 5-year recurrence rate and fewer effective treatment strategies than is the ER+ subtype [12]. The outcomes of treatments aimed at suppressing key signaling pathways are far from satisfying due to the heterogeneity of ER– tumors [13]. At present, much effort is focused on the molecular subtyping of ER– breast cancers [3, 4]. For example, molecular markers such as mTOR and Src are reportedly involved in the development of ER– tumor metastasis [14].

Although strong evidence indicates LOX promotes bone metastasis in patients with breast cancer [7], the association between LOX and prognosis in ER– breast cancer patients remained unclear. We address that issue in the present study and show the significant prognostic power of LOX in ER– breast cancer. Given that survival us usually poor among patients with high LOX expression, LOX could be a useful biomarker to stratify patients with ER– breast cancer and direct personalized therapies.

Bisphosphonates are common medications used to treat osteoporosis and may suppress bone metastasis by inhibiting LOX [7, 15]. Moreover, our analysis indicates that LOX is likely co-expressed with MMP2, COL1A1 and SPARC, all of which contribute to initiating cancer metastasis and EMT [9, 10, 16–18], and that bisphosphonates inhibit MMP2 activity [19, 20], COL1A1-driven osteoporotic fracture [21] and SPARC-stimulated EMT [22, 23]. Bisphosphonates may also inhibit breast cancer progression by decreasing stromal TGF-β excretion and inhibiting TGF-β signaling in cancer cells [24]. Based on these findings, we suggest that bisphosphonates are potentially effective chemotherapeutics for treating LOX+ ER– breast cancer patients.

Gemcitabine and cisplatin are currently used in adjuvant settings for treatment of breast cancer. Hu et al. [25] showed cisplatin plus gemcitabine could be an alternative or even the preferred first-line therapeutic strategy for patients with metastatic triple-negative breast cancer. Since LOX expression positively correlates with cisplatin and gemcitabine sensitivity, LOX+ ER– breast cancer patients may show a higher response rate to these drugs.

Inhibition of PARP is an effective strategy for suppressing cancers with BRCA1 and BRCA2 mutations. There are currently several PARP inhibitors in clinical trials aimed at evaluating their efficacy for the management of BRCA-mutated breast cancers [11]. For instance, among patients with metastatic triple-negative breast cancer, the response rate was significantly higher among patients receiving chemotherapy plus iniparib (a PARP inhibitor) than among those receiving chemotherapy alone (56% vs. 34%, *p* = 0.01) [26]. It was also reported that preoperative combined gemcitabine, carboplatin and iniparib is effective for management of early-stage triple-negative and BRCA1/2 mutation-associated breast cancer [27]. The correlation between LOX and BRCA mutation has never been reported before, but our analysis indicates that LOX expression correlates significantly with BRCA1 mutation. Since BRCA1 mutation carriers are sensitive to PARP inhibitors, some LOX+ ER– breast cancer patients may also benefit from PARP inhibitors.

In sum, our findings show that radiation, doxorubin and mitoxantrone will be less effective in patients overexpressing LOX, but that PARP inhibitors, cisplatin, trabectedin and gemcitabine may yield positive effects. A limitation of this study is that all the data presented are based on bioinformatics analyses. Still needed are wet lab experiments and well-designed clinical trials before any clinical significance can be attributed.

## MATERIALS AND METHODS

### Ethics statement

For this study, only publicly available datasets were downloaded and reanalyzed. The Research Ethics Committee of Zhejiang Provincial People's Hospital therefore waived the requirement for ethical approval.

### Genomic analysis

The Gyorffy dataset [28] (*N* = 3554) was used for survival analysis. Gene-Drug interaction data was downloaded from The Comparative Toxicogenomics Database [29] GSEA [30] and all other association analyses were performed using breast cancer data (level 3, *N* = 532) from The Cancer Genome Atlas (National Cancer Institute cancer genome atlas data portal. http://tcga-data.nci.nih.gov/tcga/findArchives.htm. Accessed September 1, 2014).

### Statistical analysis

All the data were analyzed using standard statistical tests, including independent samples *t*-tests, log rank tests and Fisher's exact tests. Values of *P* < 0.05 were considered significant. R 3.2.1 (R Foundation for Statistical Computing [http://www.r-project.org/]) and GraphPad Prism 5.01 (GraphPad Software, Inc. [www.graphpad.com]) were utilized to perform the analyses.
